# Interactive effects of gestational diabetes mellitus and maximum level of total bile acid in maternal serum on adverse pregnancy outcomes in women with intrahepatic cholestasis of pregnancy

**DOI:** 10.1186/s12884-023-05621-6

**Published:** 2023-05-08

**Authors:** Tingting Liao, Xia Xu, Yulong Zhang, Jianying Yan

**Affiliations:** 1 College of Clinical Medicine for Obstetrics & Gynecology and Pediatrics, Fujian Medical University, Fujian Maternity and Child Health Hospital, Fuzhou City, Fujian Province 350001 China; 2 Department of Obstetrics and Gynecology, Fujian Maternity and Child Health Hospital, Fuzhou City, Fujian Province 350001 China

**Keywords:** Intrahepatic cholestasis of pregnancy (ICP), Gestational diabetes mellitus (GDM), Total bile acid (TBA), Perinatal outcomes

## Abstract

**Objective:**

To study the combined effect of gestational diabetes mellitus (GDM) and maximum level of maternal serum total bile acid (TBA) on the incidence of adverse pregnancy outcomes in women with intrahepatic cholestasis of pregnancy (ICP).

**Methods:**

This was an observational study with 724 women with ICP. Perinatal outcomes were compared by the presence of GDM. Logistic regression was used to assess the independent and multiplicative interactions of GDM and maximum maternal serum TBA on adverse pregnancy outcomes. Additive interactions were calculated using an Excel sheet developed by Andersson to calculate relative excess risks.

**Results:**

The incidence of GDM in patients with ICP was 21.55%. Maternal age, pre-pregnancy weight, parity, and gravidity were positively correlated with GDM. Hypertensive disorders of pregnancy (HDP) and fetal distress rates were higher in the GDM vs. non-GDM group. There were no significant differences in biochemical outcomes (i.e., Triglyceride (TG), low density lipoprotein (LDL), alanine aminotransferase (ALT), aspartate aminotransferase (AST) and total bile acid (TBA)) between the two groups. In terms of adverse pregnancy outcomes, GDM was only associated with maximum TBA concentration for cesarean section. No additive or pairwise interactions were detected between GDM and maximum TBA concentration and HDP, PPH, preterm delivery, LGA, SGA, and cesarean section.

**Conclusion:**

GDM independently contributes to adverse pregnancy outcomes among women with ICP. However, the combined effects of GDM and maximum TBA concentration on adverse pregnancy outcomes do not appear to be multiplicative or additive.

## Background

Intrahepatic cholestasis of pregnancy (ICP), also known as pregnancy-specific liver disease, is characterized by unexplained maternal pruritus, elevated serum total bile acid (TBA) levels, and abnormal serum liver tests. Its diagnosis also involves ruling out other liver diseases that may lead to elevated bile acids [1]. The incidence of ICP varies between ethnic groups, ranging from 0.08–27.6% [2]. ICP has a multifactorial etiology with environmental, endocrine, and genetic contributions [3]. ICP is associated with various adverse pregnancy outcomes, including spontaneous preterm labour, fetal hypoxia, meconium-stained liquor, and stillbirth [4]. Moreover, severe ICP (defined as maternal serum bile acid levels > 40 µmol/L) has been reported to lead to complex pregnancy outcomes [5].

Gestational diabetes mellitus (GDM) is a common pregnancy-related metabolic diseases diagnosed when hyperglycemia first appears during pregnancy [6]. The pathogenesis of GDM is unknown. Heredity [7]and environmental [8]factors have been shown to play an important role in the occurrence of GDM, as has chronic inflammation [9], abnormal lipid metabolism [10], and insulin resistance [11]. It is worth noting that GDM has an extensive and far-reaching impact on the health of mothers and offspring [12]. Adverse pregnancy outcomes include macrosomia, neonatal hypoglycemia, and hyperbilirubinemia [13], while long-term effects are related to cardiovascular and metabolic diseases [14].

Research has established that abnormal bile acid levels can lead to glucose and lipid metabolism disorders [15]. Furthermore, a growing body of literature indicates that women with ICP are more likely to develop GDM [16]. Our study aims to explore the interactive effects of GDM and maximum TBA concentration on adverse pregnancy outcomes in women with ICP.

## Methods

### Patients

**Fig. 1:**
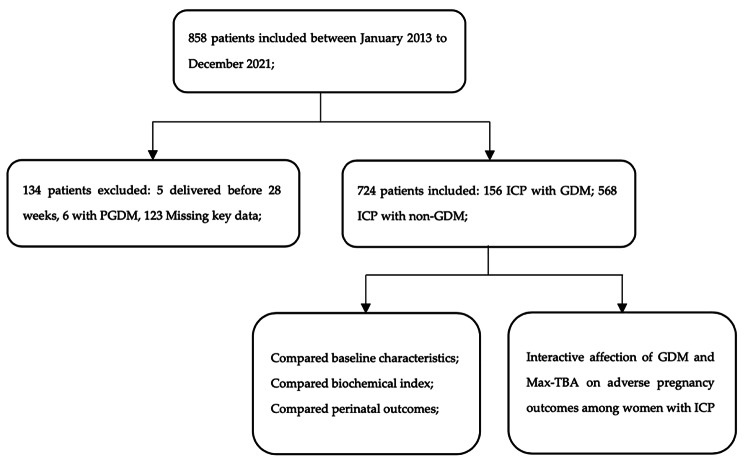
Flow of participants into the study

This retrospective and observational study included all women who were followed at the Fujian Maternity and Child Health Hospital (China) between January 2013 and December 2021, with singleton pregnancies that extended to or beyond 24 weeks of gestation. A total of 858 pregnant women with ICP were screened, of which 134 were excluded due to delivery before 28 weeks (n = 5), diagnosis of PGDM which had diabetes before pregnancy (n = 6), and missing key data (n = 123). Thus, our study included 724 women: 156 with ICP and GDM (study group) and 568 with ICP and non-GDM pregnancies (control group). ICP was diagnosed based on new-onset pruritus with a TBA level > 10 µmol/L and the absence of any additional liver diseases. The diagnostic criteria for GDM were based on the National Health and Family Planning Commission of the People’s Republic of China guidelines. When the 75 g OGTT results met or exceeded the following plasma glucose levels at the noted time-points, the women were diagnosed with GDM: 0 h, 5.1 mmol/L; 1 h, 10.0 mmol/L; and 2 h, 8.5 mmol/L. A 75 g OGTT was performed between the 24th and 28th weeks of gestation for all pregnant women who had not previously been diagnosed with diabetes. The study group consisted of all ICP pregnancies with GDM, as demonstrated by a positive OGTT status on antenatal screening and the control group consisted of the ICP pregnancies whose blood glucose was at normal level. Figure 1 shows a flow chart of study from total results to the final inclusion or exclusion.

This retrospective study involving human participants were reviewed and approved by Ethics committee of the Fujian Maternity and Children Hospital (2021KLRD631). Written informed consent for participation was not required for this study in accordance with the national legislation and the institutional requirements.

### Inclusion and exclusion criteria

The inclusion criteria of this trial were as follows: age ≥ 18 years, singleton pregnancy, and ICP status (as reflected by TBA level on antenatal screening). The exclusion criteria were as follows: patients with pre-existing or a history of medical conditions, including chronic hypertension, diabetes, or other cardiovascular, endocrinological, urogenital, gastrointestinal, autoimmune, or oncological disease; thrombophilia; multiple gestation; or in active labor or patients on ursodeoxycholic acid (UDCA) treatment were excluded from the study. Also excluded were patients who used medications that could interfere with blood glucose, such as glucocorticoids, patients with pre-gestational obesity (body mass index [BMI] > 30 kg/m^2^), and smokers.

### Outcome measurements

We reviewed the records of 724 women who had delivered their infants during the study period and recorded the following parameters: baseline characteristics of the included patients, including maternal age (years), gestational age (weeks; determined from the fetal crown–rump length), gravidity, parity, pre-pregnancy weight (kg), pre-pregnancy BMI (kg/m^2^) and gestational weight gain (kg); biochemical index before delivery of the included patients including Triglyceride (TG, mmol/L), low density lipoprotein (LDL, mmol/L), alanine aminotransferase (ALT, IU/L), aspartate aminotransferase (AST, IU/L) and total bile acid (TBA, µmol/L). TBA concentration in this study was measured by fasting maternal serum. Maximum level of TBA in maternal serum referred to the highest concentration of TBA during pregnancy, regardless of whether accept medication or not.

The main pregnancy outcomes in this study included: hypertensive disorders of pregnancy (HDP), defined as blood pressure ≥ 140/90 mmHg that occurred after 20 weeks gestation but without proteinuria [17]; preterm premature rupture of membrane (PPROM), spontaneous rupture of membranes before the onset of labor; oligohydramnios, defined as an amniotic fluid index of 5 cm or less; macrosomia, defined as a birth weight of more than 4000 g [18]; Large for gestational age (LGA) or small for gestational age(SGA), defined as a birth weight more than 90th or less than 10th percentile based on gender and gestational age [19]; liver dysfunction, mode of delivery (cesarean section or non-cesarean section); preterm delivery, defined as gestational age at delivery < 37 weeks but > 28 weeks; postpartum hemorrhage (PPH), defined as blood loss of 500 ml or more within 24 h after vaginal birth or 1000 ml or more within 24 h after cesarean section; placental weight (g), 2 h postpartum hemorrhage (mL); fetal distress, defined as a fetus suffering from insufficient oxygen supply, based on abnormal fetal heart rate and movements; gender (male or female), birth weight (g), birth height (cm).

### Statistical analyses

SPSS 19.0 (SPSS Inc., Chicago, IL) was used for statistical analyses. Continuous data (i.e., age and biochemical outcomes) are presented as means  ±  standard deviation (SD) and were analyzed using an independent t-test or a nonparametric test (Kruskal–Wallis test). Dichotomous data (e.g., sex) are presented as percentages and were compared using the χ [2] test or a nonparametric test (Fisher’s exact test). Significance was set at *P < 0 0.05*. A logistic regression model was first used to calculate the regression coefficients and covariance matrix for two factors. The data were then inputted into an Excel sheet developed by Andersson to calculate relative excess risk due to interaction (RERI), attributable proportion due to interaction (AP), interaction index (synergy index, SI), and 95% CIs [20]. The 95% CI of RERI and AP included “0” and the 95% CI of SI included “1”, indicating the absence of additive interaction.

## Results

### Baseline characteristics

Participants’ baseline characteristics are displayed in Table 1. The incidence of GDM in patients with ICP was 21.55%. Maternal age (32.24 ± 4.34 versus 29.88 ± 4.20, *P < .001*), pre-pregnancy weight (54.23 ± 8.23 versus 52.53 ± 7.83, *P = .017*), and the proportion of gravidity > 1 (64.74% versus 57.57%, *P = .011*) and multiparity (51.92% versus 41.37%, *P = .019*) were higher in the GDM group vs. non-GDM group. In contrast, gestational weight gain was lower in the GDM group (12.11 ± 5.47 versus 13.24 ± 4.87, *P = .020*). There were no significant differences in gestational age at delivery (*P = .210*) and pre-pregnancy BMI (25.59 ± 3.80 versus 25.26 ± 3.06, *P = .319*) between the GDM and non-GDM groups.

**Table 1:** Baseline characteristics of the included patients

Variables	GDM (n = 156)	Non-GDM (n = 568)	t/χ^2^/H	P value
Maternal age (mean ± SD, years)	32.24 ± 4.34	29.88 ± 4.20	6.16	0.001
Gestational age at delivery (median [IQR], weeks)	38 (37, 39)	38 (37, 39)	-1.254	0.210
Gravidity (No. [%])				
1	55 (35.26%)	241 (42.43%)	2.696	0.011
>1	101 (64.74%)	327 (57.57%)		
Parity (No. [%])				
Primiparity	75 (48.08%)	333 (58.63%)	5.538	0.019
Multiparity	81 (51.92%)	235 (41.37%)		
Pre-pregnancy weight (mean ± SD, kg)	54.23 ± 8.23	52.53 ± 7.83	2.383	0.017
Pre-pregnancy BMI (mean ± SD, kg/m^2^)	25.59 ± 3.80	25.26 ± 3.06	-0.999	0.319
Gestational weight gain (mean ± SD, kg)	12.11 ± 5.47	13.24 ± 4.87	-2.349	0.020

### Biochemical tests

The results of biochemical tests performed during pregnancy are displayed in Table 2. There were no significant differences in mean TG (3.64 ± 2.25 versus 3.42 ± 1.59, *P = .137*), LDL (2.95 versus 3.06, *P = .138*), ALT (14.20 versus 17.20, *P = .220*), AST (18.85 versus 20.15, *P = .673*), and TBA (28.84 ± 24.69 versus 27.22 ± 20.83, *P = .409*) levels between the GDM and non-GDM groups.

**Table 2:** Biochemical index before delivery of the included patients

Variables	GDM (n = 156)	Non-GDM (n = 568)	t/χ^2^/H	P value
TG (mean ± SD, mmol/L)	3.64 ± 2.25	3.42 ± 1.59	1.491	0.137
LDL (median [IQR], mmol/L)	2.95 (2.36, 3.68)	3.06 (2.45,3.89)	-1.483	0.138
ALT (median [IQR], IU/L)	14.20 (9.41,30.17)	17.20 (10.76,32.65)	1.226	0.220
AST (median [IQR], IU/L)	18.85 (14.60,30.08)	20.15 (16.23,32.76)	0.422	0.673
Max. TBA (mean ± SD, µmol/L)	28.84 ± 24.69	27.22 ± 20.83	0.826	0.409

**Note**: TG, triglyceride; LDL, low density lipoprotein; ALT, alanine aminotransferase; AST, aspartate aminotransferase; Max. TBA, maximum total bile acid

### Perinatal outcomes

Maternal outcomes are displayed in Table 3. Compared with the non-GDM group, the proportion of HDP was higher in the GDM group (15.38% versus 8.80%, *P = .016*). There were no significant differences in PPROM (16.03% versus 18.84%, *P = .420*), oligohydramnios (1.92% versus 4.23%, *P = .179*), liver dysfunction (26.92% versus 21.48%, *P = .128*), delivery mode (cesarean section: 62.18% versus 56.87%, vaginal delivery: 37.82% versus 43.13%, *P = .234*), preterm delivery (23.08% versus 17.96%, *P = .149*), PPH (0.64% versus 1.76%, *P = .311*), placental weight (572.01 ± 123.94 versus 599.62 ± 125.92, *P = .158*), and 2-hour postpartum hemorrhages (340.07 ± 161.44 versus 325.89 ± 160.37, *P = .329*) between the GDM and the non-GDM groups.

**Table 3:** Maternal outcomes of the included patients

Variables	GDM (n = 156)	Non-GDM (n = 568)	t/χ^2^/H	P value
HDP (No. [%])	24 (15.38%)	50 (8.80%)	5.778	0.016
PROM (No. [%])	25 (16.03%)	107 (18.84%)	0.649	0.420
Oligohydramnios (No. [%])	3 (1.92%)	24 (4.23%)	1.807	0.179
Liver dysfunction(No. [%])	42 (26.92%)	122 (21.48%)	2.317	0.128
Mode of delivery (No. [%])				
Cesarean section	97(62.18%)	323 (56.87%)	1.418	0.234
vaginal delivery	59 (37.82%)	245 (43.13%)		
Preterm delivery (No. [%])	36 (23.08%)	102 (17.96%)	2.079	0.149
PPH	1 (0.64%)	10 (1.76%)	1.025	0.311
Placental weight (mean ± SD, g)	572.01 ± 123.94	599.62 ± 125.92	-1.418	0.158
2 h postpartum hemorrhage (mean ± SD, mL)	340.07 ± 161.44	325.89 ± 160.37	-0.977	0.329

**Note**: HDP, hypertensive disorders of pregnancy; PROM, premature rupture of membrane; PPH, postpartum hemorrhage

Fetal outcomes are displayed in Table 4. Compared with the non-GDM group, the GDM group had higher rates of fetal distress (1.92% versus 0.35%, *P = .036*). No significant differences were found in LGA (3.21% versus 1.76%, *P = .262*), SGA (3.85% versus 5.46%, *P = .418*), gender (male: 49.36% versus 53.17%, female: 50.64% versus 46.83%, *P = .416*), birth weight (2964.22 ± 654.98 versus 3058.06 ± 549.77, *P = .071*), and birth height (48.45 ± 2.61 versus 48.22 ± 3.37, *P = .373*) between the GDM and the non-GDM groups.

**Table 4:** Fetal outcomes of the included patients

	GDM (n = 156)	Non-GDM (n = 568)	t/χ^2^/H	P value
LGA (No. [%])	5 (3.21%)	10 (1.76%)	1.259	0.262
SGA (No. [%])	6 (3.85%)	31 (5.46%)	0.655	0.418
Fetal distress (No. [%])	3 (1.92%)	2 (0.35%)	4.404	0.036
Gender				
Male	77 (49.36%)	302 (53.17%)	0.399	0.416
Female	79 (50.64%)	266 (46.83%)		
Birth weight (mean ± SD, g)	2964.22 ± 654.98	3058.06 ± 549.77	1.809	0.071
Birth height (mean ± SD, cm)	48.45 ± 2.61	48.22 ± 3.37	0.891	0.373

**Note**: LGA, large for gestational age; SGA, small for gestational age

### Interactions

The associations between GDM, maximum TBA concentration, and adverse pregnancy outcomes are shown in Table 5. Only maximum TBA concentration was associated with cesarean section.

**Table 5:** Association of GDM and maximum TBA concentration with adverse pregnancy outcomes

Variables	HDP	PPH	Preterm delivery	LGA	SGA	C-section
GDM	2.47(0.97, 6.29)	N.A.	1.36(0.61, 3.06)	1.26(0.14, 11.56)	0.47(0.10, 2.16)	1.88(0.95, 3.72)
maximum TBA concentration	1.00(0.99, 1.02)	0.978(0.93, 1.03)	1.02(1.01, 1.03)	0.97(0.93, 1.03)	0.99(0.98,1.02)	1.02(1.01, 1.03)

**Note** : Data are shown as OR (95%CI). TBA, total bile acid; LGA, large for gestational age; SGA, small for gestational age; HDP, hypertensive disorders of pregnancy; PPH, postpartum hemorrhage. For all outcomes except preterm delivery were adjusted for maternal age, gestational age, infant sex, gravity, and parity

The pairwise interaction between GDM and maximum TBA concentration on adverse pregnancy outcomes is displayed in Table 6. No pairwise interactions were detected between GDM and maximum TBA concentration and HDP, PPH, preterm delivery, LGA, SGA, and cesarean section. The additive interactions between GDM and maximum TBA concentration and adverse pregnancy outcomes are displayed in Table 7. No additive interactions were detected between GDM and maximum TBA concentration and HDP, PPH, preterm delivery, LGA, SGA, and cesarean section.

**Table 6:** Pairwise Interaction of GDM and maximum TBA concentration on Adverse Pregnancy Outcomes

Variables	HDP	PPH	Preterm delivery	LGA	SGA	C-section
GDM maximum TBA concentration	0.99(0.97, 1.01)	2.37(0,1.51)	0.99(0.98,10.2)	1.02(0.95, 1.09)	1.01(0.98, 1.05)	0.99(0.97, 1.00)

**Note** : Data are shown as OR(95%CI). TBA, total bile acid ; LGA, large for gestational age; SGA, small for gestational age; HDP, Hypertensive disorders of pregnancy; PPH, postpartum hemorrhage

**Table 7:** Additive interaction of GDM and maximum TBA concentration on Adverse Pregnancy Outcomes

Variables	RERI (95%CI)	API (95%CI)	SI (95%CI)
HDP	-0.011(-0.072,0.051)	-0.0044(-0.030,0.021)	0.99(0.95,1.04)
PPH	0.022(-0.024,0.068)	NA	0.98(0.94, 1.02)
Preterm delivery	0.0072(-0.028,0.042)	0.0051(-0.0171,0.027)	1.02(0.96,1.09)
LGA	0.0119(-0.082,0.11)	0.0096(-0.075,0.094)	1.05(0.47,2.36)
SGA	0.0068(-0.013,0.027)	0.014(-0.026,0.0549)	0.99(0.94,1.03)
C-section	-0.0042(-0.019,0.011)	-0.0022(-0.011,0.0068)	1.00(0.97,1.02)

**Note** : Data are shown as OR (95%CI). TBA, total bile acid; LGA, large for gestational age; SGA, small for gestational age; HDP, Hypertensive disorders of pregnancy; PPH, postpartum hemorrhage

## Discussion

In this population-based study, we found that GDM was associated with differences in maternal and fetal outcomes in women with ICP. We also explored the interactive effects of GDM and maximum TBA concentration on adverse pregnancy outcomes in women with ICP. We determined that the incidence of GDM in patients with ICP was 21.55%, which was similar to the 27.45% reported by Majewska [21]. We also found that maternal age, pre-pregnancy weight, parity, and gravidity were positively associated with GDM. This latter finding is novel, as many researchers have reported that age, parity, and gravidity are significantly higher among patients with GDM as compared with healthy controls [22]. ICP can be divided into mild and severe, and according to the guidelines, severe ICP is the reason for a planned cesarean section [23]. This study did not find a difference in gestational age at delivery and pre-pregnancy BMI in pregnant women with vs. without GDM. Further research remains to be done.

This study determined that the proportion of HDP and fetal distress was higher among women with GDM than those without GDM. Further, no significant between-group differences were found in PPROM, oligohydramnios, liver dysfunction, delivery mode, preterm delivery and PPH, placental weight, 2-hour postpartum hemorrhage, LGA, SGA, gender, birth weight, and birth height. Axelsen et al. found that significantly more women with GDM and ICP developed preeclampsia during pregnancy as compared with women with only GDM [24]. Similarly, a meta-analysis conducted by Zhang et al. determined that ICP significantly increased the risk of both PE and GDM [25]. These findings are consistent with the results of our study.

We did not find any significant differences in biochemical outcomes (i.e., TG, LDL, ALT, AST and TBA) between the GDM and non-GDM groups. In terms of adverse pregnancy outcomes, GDM was only associated with maximum TBA concentration for cesarean section. No additive or pairwise interactions were detected between GDM and maximum TBA concentration and HDP, PPH, preterm delivery, LGA, SGA, and cesarean section. These findings are consistent with the results of Majewska and others, which did not find any significant correlations between the blood glucose levels of pregnant women with ICP and ALT, AST, and perinatal outcomes [21]. However, Majewska et al. believe that the decrease in serum TBA levels in patients with ICP is related to the presence of GDM [21]. In contrast, our study suggests that there are no significant differences in the serum TBA levels of women with and without GDM. In a separate study, Gao et al. found that the serum levels of 8 bile acids were elevated among women with GDM as compared with healthy controls [26]. Further, Jin et al. reported that compared with a healthy control group, every increase in TG level in the third trimester of pregnancy increased the risk of GDM, while increasing LDL levels reduced the risk of GDM [27]. Future studies should explore the effects of GDM on the blood lipid levels of women with ICP.

There are some limitations of our study. First, as a retrospective design, confirmation of causal association is limited. Second, there is no detailed analysis of the specific treatment methods of pregnant women with GDM. Third, the measurement of TBAs is very method-dependent and can also be negatively influenced by drugs such as UDC. Also, in our cohort, we did not divide ICP into severe group (TBA ≥ 100 µmol/l) and mild group (TBA < 100 µmol/l) for further study. Although these limitations, our study is the first time to study the combined effect of GDM and maximum level of maternal serum TBA on the incidence of adverse pregnancy outcomes in women with ICP. The second strength is that we adjusted for the potential mediating effect and considered results reliable.

## Conclusion

GDM independently contributes to adverse pregnancy outcomes among women with ICP. However, the combined effects of GDM and maximum TBA concentration on adverse pregnancy outcomes do not appear to be multiplicative or additive.

## Data Availability

The datasets used and analyzed during the current study are available from the corresponding author on reasonable request.

## Abbreviations

ALT: Alanine aminotransferase
AST: Aspartate aminotransferase
BMI: Body mass index
GDM: Gestational diabetes mellitus
ICP: Intrahepatic cholestasis of pregnancy
LDL: Low-density lipoprotein
TBA: Total bile acid
TG: Triglyceride
